# Sulfur-Containing Compounds: Natural Potential Catalyst for the Isomerization of Phytofluene, Phytoene and Lycopene in Tomato Pulp

**DOI:** 10.3390/foods10071444

**Published:** 2021-06-22

**Authors:** Lulu Ma, Cheng Yang, Xin Jiang, Qun Wang, Jian Zhang, Lianfu Zhang

**Affiliations:** 1 School of Food Science and Technology, Jiangnan University, Wuxi 214122, China; 6180112053@stu.jiangnan.edu.cn (L.M.); cheng.yang@jiangnan.edu.cn (C.Y.); 6170111022@stu.jiangnan.edu.cn (X.J.); 6190111077@stu.jiangnan.edu.cn (Q.W.); zhangjian0411@163.com (J.Z.); 2 The Food College, Shihezi University, Shihezi 832003, China

**Keywords:** lycopene, phytofluene, phytoene, sulfur-containing compounds, isomerization

## Abstract

The effects of some sulfur-containing compounds on the isomerization and degradation of lycopene, phytofluene, and phytoene under different thermal treatment conditions were studied in detail. Isothiocyanates such as allyl isothiocyanate (AITC) and polysulfides like dimethyl trisulfide (DMTS) had the effect on the configuration of PTF (phytofluene), PT (phytoene), and lycopene. The proportion of their naturally occurring *Z*-isomers (*Z*1,2-PTF and 15-*Z*-PT) decreased and transformed into other isomers including all-trans configuration, while *Z*-lycopene increased significantly after thermal treatment, especially for 5-*Z*-lycopene. The results showed that increase in heating temperature, time, and the concentration of DMTS and AITC could promote the isomerization reaction effectively to some extent. In addition, 15-*Z*-PT and the newly formed *Z*4-PTF were the predominant isomers in tomato at the equilibrium. Unlike the lycopene, which degraded significantly during heat treatment, the isomers of PTF and PT were stable enough to resist decomposition. Moreover, the isomerization of three carotenoids was enhanced, and the bioaccessibility of lycopene increased significantly with the addition of shii-take mushroom containing sulfur compounds, while there was no positive effect observed in that of PTF and PT.

## 1. Introduction

Tomato, as one of the most widely cultivated crops in the world, is rich in phenolic compounds, vitamin C, carotenoids, amino acids, etc. [1]. Tomato and tomato-based products play an important role in diet because of their unique flavor and high nutritional value [2]. Numerous epidemiological studies showed that tomato consumption can reduce the risk of many cancers, including breast and prostate cancer [3,4], as well as chronic diseases such as cardiovascular disease [5] and diabetes [3]. 

Carotenoids are the most important nutrients in tomato including lycopene, phytofluene (PTF), phytoene (PT), etc. Lycopene is the main carotenoid and responsible for the red color of tomatoes. However, Basu et al. [6] pointed out that the health benefits of consuming tomato and its products may the synergies between lycopene and other ingredients, not only lycopene. As a matter of fact, PTF and PT are also important to human health. In an assessment in Luxembourg, the daily intake of PTF and PT contributed 16% of total daily carotenoid intake, which was higher than lycopene [7]. Particularly, PTF and PT are strongly present in their *cis*-isomers in natural plants. Due to their naturally existing *cis*-isomers, the bioavailability and bioaccessibility of them are much higher than lycopene in the same food matrices [8,9]. In addition, PTF and PT are proved to have health-promoting actions, such as antioxidant [10] and anti-inflammatory properties along with a reduced risk of various diseases [11]. Besides, they can protect skin against UV damage as their maxima absorption was in the ultraviolet region [12]. However, PTF and PT were less concerned in tomato compared with lycopene.

More than 90% of lycopene present in natural plants is all-*E*-lycopene [13], however, there are about 50% of the total lycopene is *Z*-isomers in human serum and tissues [14]. Lycopene can form many geometric isomers because of the carbon−carbon conjugated double bonds. The most common *Z*-lycopene found in processed tomato matrices including 5-*Z*-, 9-*Z*-, and 13-*Z*-lycopene [15]. Many studies showed that the isomerization of all-*E*-lycopene to higher bioactive *Z*-lycopene happened during tomato processing [16,17,18,19]. More recently, it was found that the addition of some ingredients into tomato showed a great significant to the *Z*-isomerization of lycopene. Honda, et al. found that some traditional seasonings [20] (e.g., jerk sauce, tapenade, aioli) and food ingredients [21] (e.g., Allium sp., shiitake mushroom) can promote the *Z*-isomerization reaction of lycopene with thermal treatment. Besides, Yu, et al. [22] combined onions and tomato sauce to promote *Z*-isomerization of lycopene and increased accessibility of total lycopene. Further research showed that some sulfur-containing compounds, e.g., polysulfides, isothiocyanate, and carbon disulfide, in food enhanced the thermal *Z*-isomerization. Honda, et al. [23] also discussed the isothiocyanates and polysulfides on the *Z*-isomerization and decomposition of lycopene, β-carotene, and astaxanthin standard. However, most of these studies focused on the degradation and isomerization of lycopene in tomato and ignored the change of colorless carotenoids with high nutritional value.

In the case of PTF and PT, they are strongly present in their cis-isomers in natural plants. PT major presented as 15-*Z*-PT, which is synthesized by two molecules of geranylgeranyl diphosphate (C20), and PTF exists as a mixture of several isomers in plants [11]. However, most research just focused on the change of total content and ignored the isomerization of them during thermal treatment. Graziani, et al. [24] found that there was no obvious change in the content of PTF and PT after heating at 100 °C within 4 h. In the study by Cooperstone et al. [25], the content of PTF and PT unchanged in tangerine tomato after heating at 100 °C within 180 min. However, Mapelli–Brahm et al. [26] reported that the PTF and PT in orange juice decreased significantly after pasteurizing. There was less information about the isomerization of PTF and PT in tomato and the stability of their isomers during food processing. Moreover, data on their isomeric profile with sulfur-containing compounds during thermal treatment and the bioaccessibility of different isomers were scarce. Thus, the objective of this study was to investigate the effect of different sulfur-containing compounds on the isomerization and degradation of PTF, PT, and lycopene in tomato-oil under different thermal conditions, to help forecast their expected isomeric profiles in food processing. In addition, we combined tomato with shii-take mushroom which containing sulfur compounds [21,27] such as dimethyl trisulfide, dimethyl disulfide, etc., to investigate the effect on isomerization and bioaccessibility of PTF, PT, and lycopene. The results will benefit the food processers and provide information on developing tomato products rich in PTF, PT, and lycopene.

## 2. Materials and Methods

### 2.1. Chemicals and Reagents

The cherry tomatoes (*Lycopersicon esculentum var. cerasiforme*) *IVF3535* were provided by Xinjiang Guannong Fruit &Antler CO., LTD.; extra virgin olive oil and fresh shii-take mushrooms were purchased at the local supermarket; all-*E*-lycopene was purchased from Huabei pharmaceutical factory; phytofluene and phytoene(a mixture of *E/Z* isomers) were purchased from Carotenature, Switzerland; methanol, acetonitrile, and methyltert-butyl ether (MTBE) for chromatography were HPLC grade purchased from Oceanpak, Sweden; ethyl acetate, hexane, methanol, and acetone for extraction were analytical grade purchased from Sinopharm, China. Methyl isothiocyanate (MITC), butyl isothiocyanate (BITC), diallyl disulfide (DADS) and dimethyl trisulfide (DMTS) were purchased from Aladdin, China, and allyl isothiocyanate (AITC) and diallyl trisulfide (DATS) were purchased from J&K Scientific, China.

### 2.2. Effect of the Types of Sulfur-Containing Compounds on the Isomerization and Degradation of PTF, PT, and Lycopene

Different types of isothiocyanates (MITC, BITC, AITC) and polysulfides (DADS, DATS, DMTS) were first dissolved in olive oil at a concentration of 20 mg/g, and then 5% olive oil with or without (control group) sulfur-containing compounds was blended into tomato pulp as a mediator of the *E/Z*-isomerization. The final concentration for isothiocyanates and polysulfides in tomato puree-olive oil mixture was 1 mg/g. Obtained mixture was added into a 20 mL screw-capped glass bottle and air was purged by nitrogen gas. The mixture was then heated at 80 °C for 1 h in a water bath. After thermal treatment, samples were immediately cooled down and stored at −20 °C until analysis.

### 2.3. Effect of the Concentration of DMTS and AITC on the Isomerization and Degradation of PTF, PT, and Lycopene

One of the polysulfides and isothiocyanates each—DMTS and AITC—were selected to investigate the effect of concentration on the isomerization and degradation during thermal treatment. Briefly, DMTS and AITC were both dissolved in olive oil in the concentration of 4, 10, 20, 40, 80, and 100 mg/g, respectively, then 5% olive oil was blended into tomato pulp. The concentration of DMTS or AITC in mixture was 0.2, 0.5, 1, 2, 4, and 5 mg/g in the end. The mixture was then put into a glass bottle, purged of air by nitrogen gas, and heated at 80 °C for 1 h in a water bath. After thermal treatment, samples were immediately cooled down and stored at −20 °C until analysis.

### 2.4. Effect of the Reaction Temperature on the Isomerization and Degradation of PTF, PT, and Lycopene

One of the isothiocyanates (AITC) and polysulfides (DMTS) each were chosen for further study. DMTS and AITC were dissolved in olive oil at a concentration of 20 mg/g, respectively, and then 5% olive oil was blended into tomato pulp. The final concentration of DMTS and AITC was 1 mg/g mixture. Then the mixture was heated at different temperature (40 °C, 60 °C, 80 °C, 100 °C, 120 °C) for 1 h. After the thermal treatment, samples were immediately cooled down and stored at −20 °C until analysis.

### 2.5. Effect of the Reaction Time on the Isomerization and Degradation of PTF, PT, and Lycopene 

DMTS and AITC were dissolved in olive oil at a concentration of 20 mg/g, and then 5% olive oil was blended into tomato pulp. The final concentration of DMTS and AITC was 1 mg/g mixture. Then the mixture was heated at 80 °C for 0, 10, 20, 30, 60, 90, and 120 min. After the thermal treatment, samples were immediately cooled down and stored at −20 °C until analysis.

### 2.6. Preparation of the Tomato and Shii-Take Mushroom Mixture

Different proportion (10%, 20%, 30%, 40%, 50%) of shii-take mushroom and 5% olive oil were added into the tomato pulp. The mixture was homogenized by a food processor and then transferred into a 50 mL screw-capped glass bottle and air was purged by nitrogen gas. The mixture was then heated at 80 °C for 1 h in a water bath. After thermal treatment, samples were immediately cooled down and stored at −20 °C until analysis.

### 2.7. In Vitro Digestion Procedure

The in vitro digestion was developed by Yu, et al. [28] with minor modifications. Two g mixtures were weighed into a 50 mL conical flask. 20 mL 0.9% NaCl and 2 mL of porcine pepsin solution (32 mg/mL in 0.1 M HCl) were added. The pH was adjusted to 2.0 with 2.5 M HCl, and then the mixture was incubated at 37 °C for 1 h at 300 rpm to mimic the gastric digestion. Finally, 9 mL of porcine bile (24 mg/mL in 0.1 M NaHCO3) and 4 mL of pancreatin (12 mg/mL in 0.1 M NaHCO_3_) were added, and the pH was adjusted to 7.0 with 0.25 M NaOH. Mixtures were further incubated at 37 °C for 2 h at 300 rpm to mimic digestion. The digesta were centrifuged at 4 °C for 1 h at 5000 g, and the aqueous micellar phase was collected and passed through 0.22 µm cellulose esters filters (Millipore) and stored at −20 °C until analysis.

### 2.8. HPLC Analysis and Identification of Lycopene, Phytofluene, and Phytoene Isomers

Isomers of lycopene, PTF, and PT were analyzed by reversed-phase HPLC equipped with a diode array detector (Alliance 2695; Waters Corp., Milford, MA, USA). The method was developed by Cooperstone et al. [25]. The carotenoids were separated using a C30 reversed phase column (150 × 4.6 mm, 3 µm; YMC Co, Kyoto, Japan). For the quantification of lycopene, PTF and PT isomers were performed by peak area at 471, 348, and 286 nm, respectively. The identification of lycopene, PTF, and PT isomers was conducted by comparison of their retention times with that of standards and spectroscopic features (Q-ratio for lycopene, %III/II for PTF, and PT [29]). The quantification of all-*E*-lycopene, PTF, and PT isomers was determined by their external standard curve, and *Z*-lycopene was quantified with the same calibration curve of all-*E*-lycopene standards.

### 2.9. Extraction of Carotenoids 

Carotenoids extraction from the tomato [25]: briefly, about 5 g of the tomato pulp were added into triangle beaker with 20 mL extraction solvent: n-hexane: methanol: acetone (2:1:1, *v/v*/*v*), and then stirred 20 min under the dim light to prevent the degradation of carotenoids. The supernatant was collected by vacuum filtering, and filtered residue was repeated extraction two times until the color faded. The supernatant was combined together and the organic phase which containing carotenoids was separated with separating funnel, and then concentrated by rotary evaporator at 35 °C. The concentrated extraction was dissolved in ethyl acetate and diluted with methanol: MTBE (1:1, *v/v*) to a proper concentration.

Carotenoid extraction from the aqueous micellar phase: 3 mL of the micellar was extracted three times with 5 mL of hexane and methanol (1:1 *v/v*). The organic phase was collected and evaporated to dryness under nitrogen and dissolved in 100 µL methanol: MTBE (1:1, *v/v*) for the HPLC quantification.

### 2.10. Statistical and Data Analysis

All experiments in this work were carried out in triplicate; the values were presented as means values ± standard deviation (SD). The statistical significance (*p* < 0.05) was determined by Tukey’s multiple comparison test.

## 3. Results and Discussion

### 3.1. Profile of Phytofluene, Phytoene, and Lycopene Isomers after Thermal Treatment

**Figure A1:**
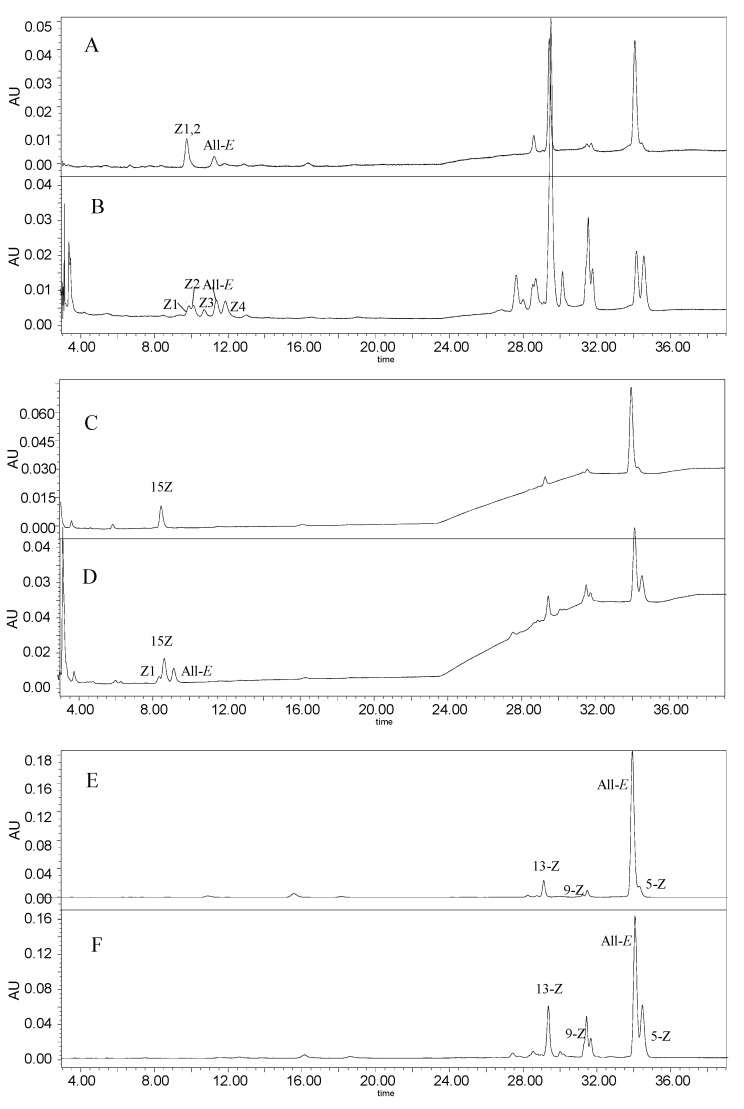
HPLC chromatogram of phytofluene at 348 nm (**A**,**B**), phytoene at 286 nm (**C**,**D**), and lycopene at 471 nm (**E**,**F**) before (**A**,**C**,**E**) and after (**B**,**D**,**F**) heating at 80 °C for 1 h with DMTS.

**Figure A2:**
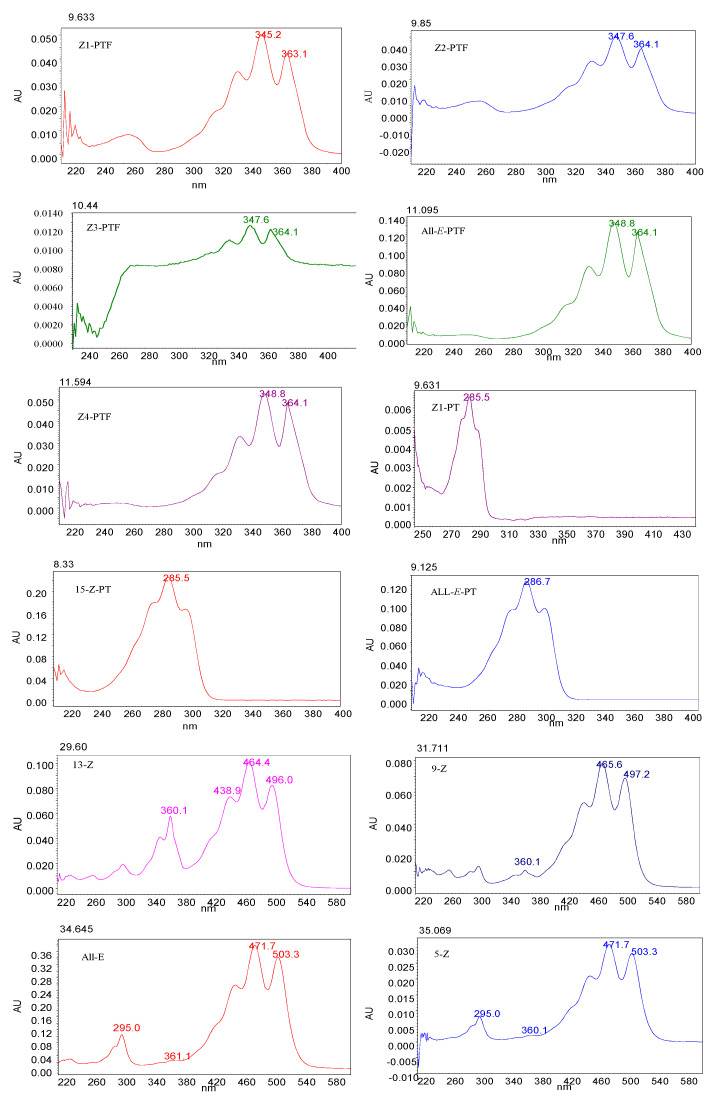
Spectra in mobile phase of main geometric isomers of PTF, PT, and lycopene.

After thermal treatment with sulfur-containing compounds, the profile of PTF, PT, and lycopene in tomato pulp changed markedly as shown in Figure A1. The isomers of PTF, PT, and lycopene were identified by their chromatographic elution order compared with that of standards, and UV–V are spectral features (as illustrated in Figure A2), λmax, %III/II, and Q ratio [29,30,31,32]. The details are shown in Table 1. The spectra of cis-isomers exhibit a reduction of fine structure relative to the all-*E*-isomer, and therefore, we identified the peak of all-*E*-PTF and all-*E*-PT as their large number of %III/II [29,32].

For PTF, we only detected two main peaks in raw tomato pulp (as illustrated in Figure A1)*,* and two new isomers generated after heat treatment. *Z*1-PTF and *Z*2-PTF cannot separate from each other, as reported by Yu, et al. [28], so we quantified them as *Z*1,2-PTF as a whole. The *Z*1,2-PTF was the most abundant isomer, which accounted for 78% of PTF in raw tomato pulp. Meanwhile, only one peak of PT was detected and identified as 15-*Z*-PT because it was the major geometrical isomer in carotenogenic organisms [33]. Interestingly, after thermal treatment, two new isomers (*Z*1-PT and all-*E*-PT) generated in tomatoes, as reported in [32]. Moreover, lycopene was represented in mainly all-*E* configuration and in raw tomato pulp. For instance, all-*E*-lycopene accounted for 80.1–86.5% and the proportion of 5-*Z*-lycopene was about 6%, the others isomers were represented in cis-configuration, which was in accordance with the report of Colle et al. [34] After heating with the sulfur-containing compounds, the peak area of *Z*-lycopene increased obviously, which indicated that higher amount of *Z*-lycopene were produced through the trans-cis isomerization of all-*E*-lycopene by the catalysis of sulfur-containing compounds. Yu et al. [22] also found that it was DADS in onion which were responsible for the increased *Z*-lycopene during heating tomato puree with onion.

### 3.2. Effect of Sulfur-Containing Compounds Types on Isomerization and Degradation of Lycopene, Phytoene, and Phytofluene

The isomerization and degradation of three different carotenoids in tomato pulp with effect of six types of sulfur-containing compounds after heating at 80 °C for 1 h was investigated. The content of PTF, PT, and lycopene was 22.9 µg/g, 13.0 µg/g, and 142.4 µg/g, respectively, in the raw tomato pulp. In the control group, the contents of total lycopene, PT, and PTF and the ratios between isomers remained the same with those in raw tomato pulp after heating with olive oil alone at 80 °C for 1 h. Colle et al. [35] also reported that there was no significant isomerization and degradation of lycopene below 100 °C, and the results reported by Cooperstone et al. [25] indicate that PT and PTF were stable enough under 100 °C with or without lipids despite being present in cis-configuration.

The ratio of each *Z*-isomer between three carotenoids changed obviously once the six sulfur-containing compounds were added as catalyst (as illustrated in Table 2 and Table 3). As for PTF, the proportion of natural occurring *Z*1,2-PTF decreased after the sulfur-containing compounds added. Meanwhile, two new *Z*-peaks, *Z*3- and *Z*4-PTF, could be observed. In the research of Meléndez–Martínez et al. [32], they separated six geometrical different isomers of PTF from a natural extract of lycopene-rich tomato. In a comparison of the effects of isothiocyanates and polysulfides, the ratio between PTF isomers was different. In the case of isothiocyanates, the proportion of all-*E*-PTF increased slightly; *Z*1,2-PTF mainly converted to *Z*3- and *Z*4-PTF. For example, when BITC was added, the proportion of *Z*1,2-PTF decreased to 31.3% from 76.5%, and all-*E*-PTF was about 5% higher than that of the control group. However, when polysulfides were used, the proportion of *Z*1,2-PTF and all-*E*-PTF were significantly decreased (*p* < 0.05) compared to that of the control group.

The same phenomenon was observed for PT. The proportion of 15-*Z*-PT decreased significantly (*p* < 0.05), while all-*E*-PT increased with the addition of catalyst. Moreover, the catalytic ability of polysulfides to PT was stronger than isothiocyanates because a new peak *Z*1-PT could be observed with the effect of polysulfides. Unlike the decomposition of lycopene, there was little degradation of total PTF and total PT in tomato pulp, which indicated that the new formed isomers of PTF and PT were stable enough to resist the degradation with the influence of heating and catalysis; this could be explained by the protection of tomato matrix. In the study of Lu et al. [30], there was not any change between the content of PTF and PT isomers in orange juice cv. Cara Cara after thermal treatment, while the content decreased obviously in simulated system. 

In the case of lycopene, the profile of generated *Z*-isomers was similar between isothiocyanates and polysulfides; all-*E*-lycopene transformed into *Z*-lycopene especially to 5-*Z*-lycopene. DMTS exhibited a higher performance in isomerization, total *Z*-isomers (accounted for 60.2%) increased by 51.2% compared with that of the initial one, of which 5-*Z*-lycopene accounted for 21.7%, almost two times higher than 9-*Z*-lycopene. The increasing formation of 5-*Z*-lycopene could be illustrated by its higher thermal stability [22]. Compared with control group, the retention rate of lycopene decreased about 80%–86% due to the formation of unstable *cis* structure with the effect of isothiocyanate or polysulfides. The mechanism of the conversion of all-*E*-carotenoids to *Z*-isomers by sulfur-containing compounds may illustrated by the thiyl radicals produced by disulfide bound [22,23]; while the isothiocyanate could promote the isomerization with it’s eletrophilicity, which works like iron(III) chloride [23]. Among isothiocyanates, the improvement of total *Z*-lycopene proportion by DADS was less than those of DATS and DMTS, which indicated that the number of disulfide bound played an important role on the isomerization of PT, PTF, and lycopene. Honda, et al. [23] also found that the *Z*-isomerization efficiency of lycopene was related to the length of disulfide bound. This may be related to the disproportionate reaction of dialkyl polysulfides at high temperatures. For instance, disproportionation/degradation of DMTS could form DMDS [36], which can further enhance the isomerization reaction.

DMTS and AITC were chosen to use in the following experiments since they were the most effective catalyst for the isomerization of carotenoids in this study and they are commonly found in vegetables and seasonings.

### 3.3. Effect of Concentration of DMTS and AITC on Isomerization and Degradation of PTF, PT, and Lycopene

**Figure 1:**
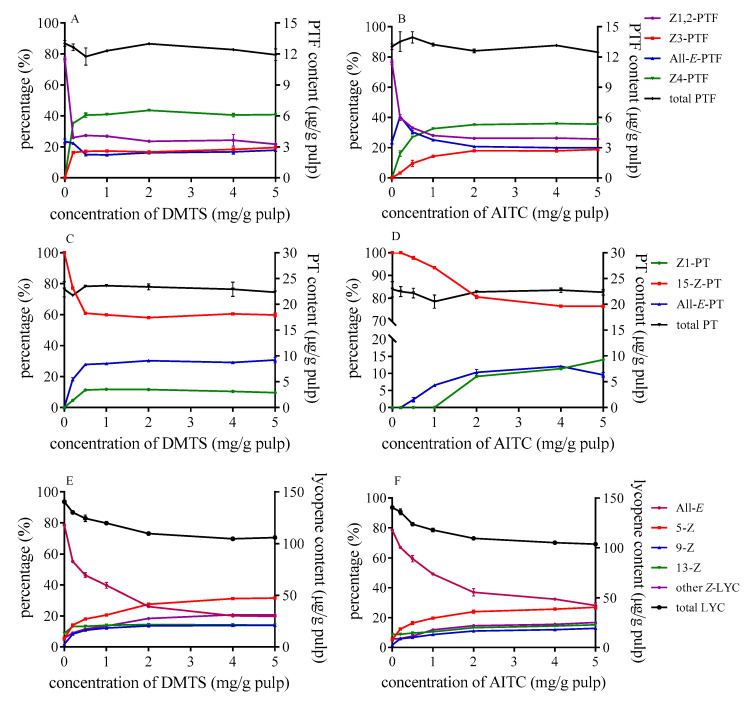
Effect of concentration of DMTS (**A**,**C**,**E**) and AITC (**B**,**D**,**F**) on isomerization and degradation of PTF (**A**,**B**), PT, (**C**,**D**) and lycopene (**E**,**F**); DMTS: dimethyl trisulfide; AITC: allyl isothiocyanate; PTF: phytofluene; PT: phytoene.

The changes in the ratio of each isomer and the content of three carotenoids in tomato pulp with different concentration of DMTS and AITC are shown in Figure 1. The proportion of *Z*-lycopene rose in tandem with an increasing number of catalysts. Newly formed *cis* configurations are mainly presented by 5-*Z*-lycopene, as it is more stable than other isomers. When DMTS was added to tomato pulp as catalyst, the ratio of total *Z*-isomers was higher than that of AITC at the same concentration, which indicated that the *Z*-isomerization efficiency of DMTS was stronger than that of AITC. For example, the proportion of all-*E*-lycopene was dropped to 20% with DMTS at a concentration of 5 mg/g (as illustrated in Figure 1E), which was less than that of AITC (29%) (as illustrated in Figure 1F) under the same condition. Moreover, the proportion of each lycopene isomer was barely changed when the concentration exceeded 2 mg/g tomato pulp. Meanwhile, the content of total lycopene decreased as the quantity of catalysts increased. The total lycopene content in the raw tomato pulp was 142.4 µg/g and decreased to 105.8 µg/g and 107.8 µg/g when 5mg/g tomato pulp DMTS and AITC added, respectively.

For PTF, the isomerization occurred even at a lower concentration of 0.2 mg/g with DMTS. Then, with the increase of catalyst concentration, the proportion of each isomer showed no significant changes. However, the proportion of isomers equilibrated at the concentration of 2 mg/g in the case of AITC (as illustrated in Figure 1B). The predominant isomer was Z4-PTF and accounted for 41% at the equilibrium, while *Z*1,2-PTF decreased to 22% with the effect of DMTS, and the proportions of all-*E*-PTF showed little change compared with those in raw tomato pulp. Several studies showed that the isomers of PTF could transform to each other during thermal treatment [30], and in the study by Meléndez–Martínez, et al. [32], the last eluted peak of PTF was the main isomers after thermal treatment with iodine. We speculated that *Z*4-PTF might be the most stable geometric isomer among the five geometric isomers under catalysis. In the case of PT, as the quantity of catalysts increased, 15-*Z*-PT decreased but all-*E*-PT and *Z*1-PT increased. The changes in the relative proportion of PT isomers were inapparent compared with PTF. 15-*Z*-PT was still the main isomers after heating. When DMTS was added as the catalyst (as illustrated in Figure 1C), the proportion of 15-*Z*-PT at the equilibrium was about 60%. The effect of AITC was weaker than DMTS, and *Z*1-PT cannot form when the concentration is less than 1 mg/g. Besides, the total PT and PTF hardly decomposed with the increased concentration of catalysts.

### 3.4. Effect of Reaction Temperature on Isomerization and Degradation of PTF, PT, and Lycopene

**Figure 2:**
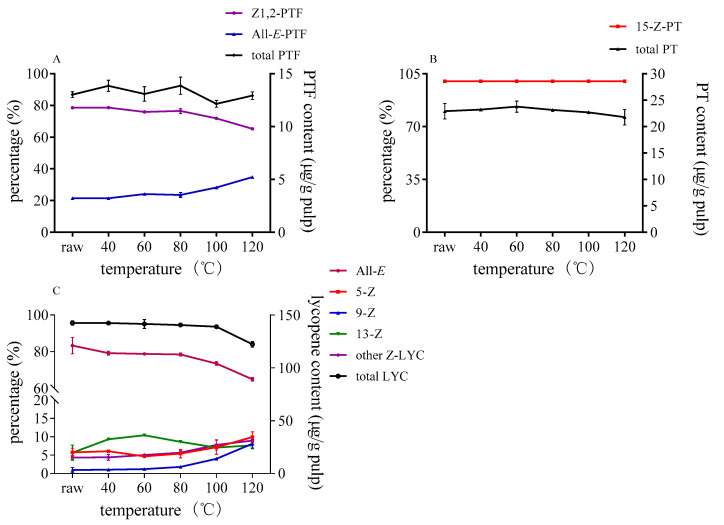
Effect of reaction temperature on isomerization and degradation of PTF (**A**), PT (**B**), and lycopene (**C**) without sulfur-containing compounds.

Carotenoids were significantly affected by temperature during processing. The changes of three carotenoids in control group without catalysts under different heating temperature are shown in Figure 2. Lycopene was stable enough under 100 °C, and the ratio of lycopene isomers and the remaining content hardly changed. However, lycopene started to degrade, and the proportion of all-*E*-lycopene decreased to 65% when the temperature reached 120 °C. Some data showed that addition of olive oil to tomato puree could increase *Z*-lycopene upon heating at 120 °C [37]. Concerning PTF, the proportion of all-*E*-PTF was slightly increased over 80 °C, and there were no newly isomers formed under 120 °C in control group. As for PT, 15-Z-PT hardly changed with the temperature rise.

**Figure 3:**
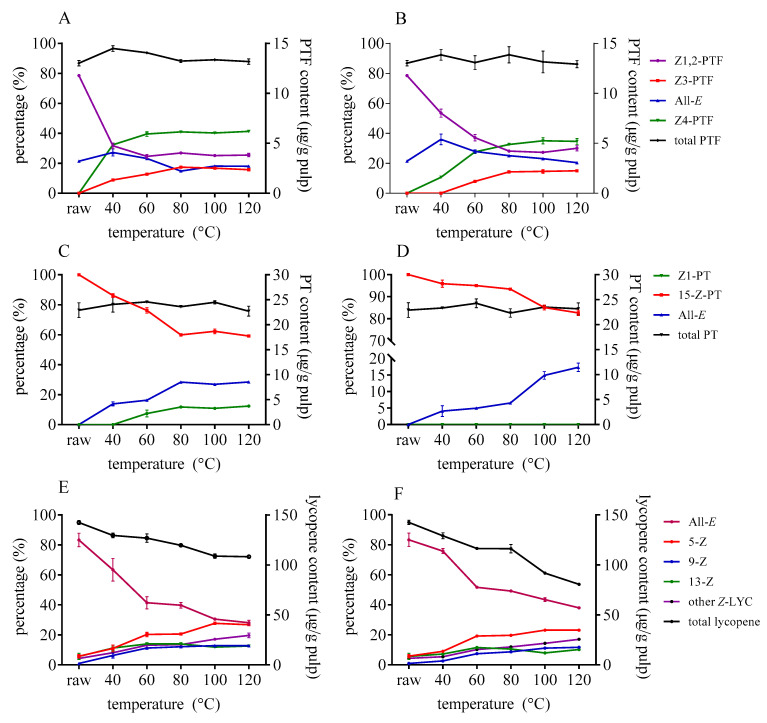
Effect of heating temperature on isomerization and degradation of PTF (**A**,**B**), PT (**C**,**D**), and lycopene (**E**,**F**) with DMTS (**A**,**C**,**E**) and AITC (**B**,**D**,**F**).

The isomerization of lycopene could happen even at a lower temperature of about 40 °C when catalysts are added (as illustrated in Figure 3). As the temperature rose, the ratio of *Z*-lycopene increased significantly. Besides, the decomposition of total lycopene progressed as the *Z*-isomerization occurred. Similarly, the isomerization efficiency of DMTS was stronger than that of AITC under the same temperature. Different from lycopene, there was no significant degradation of PTF and PT as the temperature rose, which indicate that the isomers of PTF and PT remain stable within 120 °C. Once the catalysts were added, the content of *Z*1,2-PTF decreased, while *Z*3- and *Z*4-PTF accumulated when temperature increased. *Z*1,2-PTF was still the predominant isomer at lower temperature; however, the ratio of *Z*4-PTF gradually increased as the temperature rose to 120 °C. The content of *Z*4-PTF took up about 40% of total PTF in the case of DATS after heating to 120 °C. As for PT, the content of 15-*Z*-PT significantly decreased and converted to all-*E*- and *Z*1-PT as the temperature rose. The higher the temperature was, the more *Z*1- and all-*E*-PT accumulated by DMTS (as illustrated in Figure 3C). In the case of AITC, *Z*1-PTF cannot be formed, even at a higher temperature when the concentration was 1 mg/g tomato pulp.

### 3.5. Effect of the Reaction Time on Isomerization of Lycopene, Phytoene, and Phytofluene 

**Figure 4:**
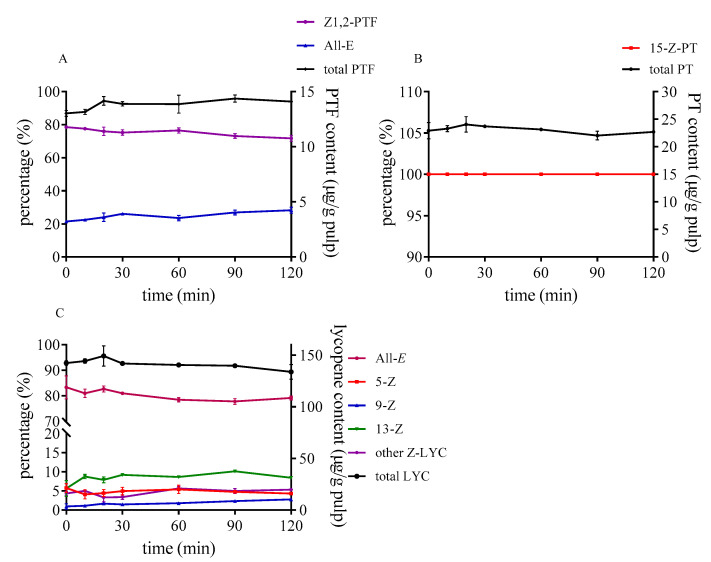
Effect of reaction time on isomerization and degradation of PTF (**A**), PT (**B**), and lycopene (**C**) without sulfur-containing compounds.

During heating tomato pulp at 80 °C from 0 to 120 min, the degradation and isomerization hardly occurred in these three carotenoids without sulfur-containing compounds (as illustrated in Figure 4). Only the proportion of 13-*Z*-lycopene slightly increased, which was probably because of its lower rotational barriers, as opposed to that of other *Z*-lycopene [38]. PTF and PT were stable within 120 min under 80 °C as well. Cooperstone et al. [25] also found that PTF and PT were unchanged when tomato sauce was processed at 100 °C for 180 min with fat.

**Figure 5:**
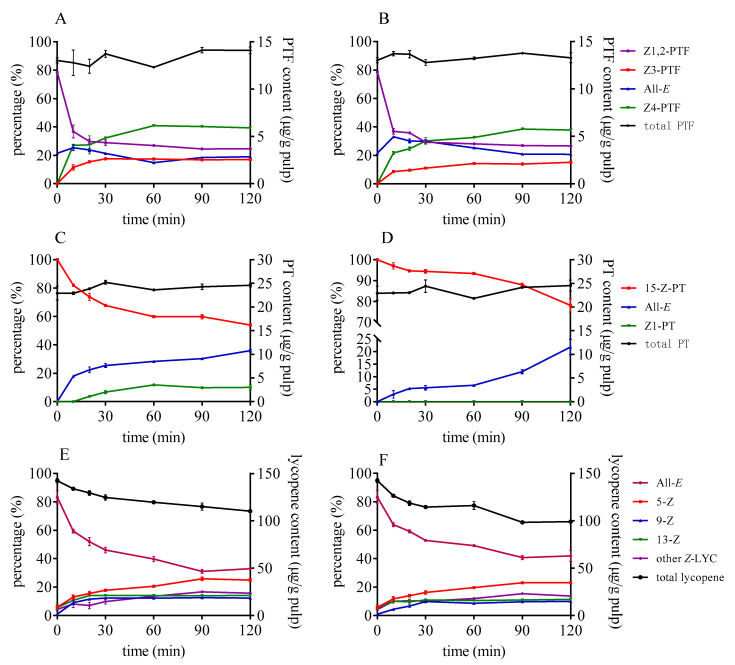
Effect of heating time on the isomerization and degradation of PTF (**A**,**B**), PT (**C**,**D**), and lycopene (**E**,**F**) with DMTS (**A**,**C**,**E**) and AITC (**B**,**D**,**F**).

As shown in Figure 5, Z-isomerization of lycopene intensified as reaction time went on when DMTS and AITC were used. The proportion of all-*E*-lycopene decreased to 33–47% after 2 h at 80 °C, and the retention rate of lycopene decreased simultaneously. The remaining lycopene decreased by 25% when AITC was added compared to that of the control group. In the case of PTF and PT, the original *Z*-isomers of PTF and PT that existed in tomato were decreased as time progressed. After heating for 60 min, the proportion between PTF isomers hardly changed, and *Z*4-PTF was the dominant one. Meanwhile, the reduction of 15-*Z*-PT was more moderate than that of *Z*1,2-PTF after heat treatment. Specially, *Z*1-PT cannot be generated when AITC is used, and all-*E*-PT decreased to 78% after heating for 120 min.

### 3.6. The Proflies and Bioaccessibility of the Lycopene, Phytoene, and Phytofluene Isomers in Tomato Heated with Shii-Take Mushroom

**Figure 6:**
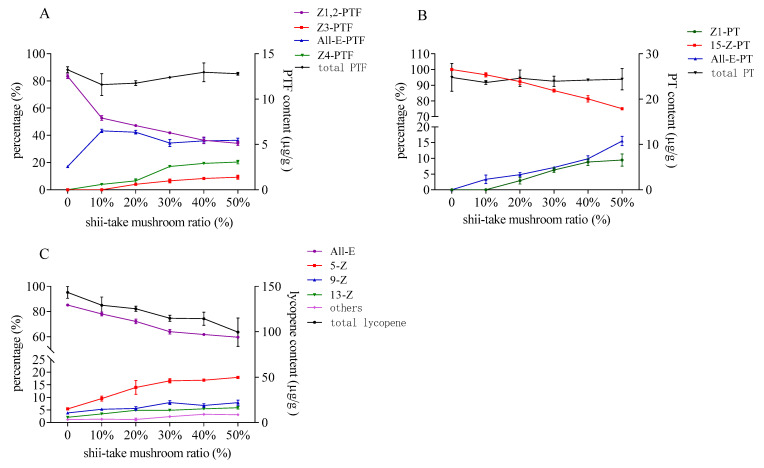
Contents and proportions of phytofluene (**A**), phytoene (**B**), and lycopene (**C**) in tomato with different addition of shii-take mushroom.

The isomer constitution and the content of PTF, PT, and lycopene after heat treatment in tomato and shii-take mushroom mixture were showed in Figure 6. The configuration of the three carotenoids changed as act in 3.2 which indicated that the sulfur- containing compounds in shii-take mushroom also had the catalysis effect on PTF, PT, and lycopene. As the added shii-take mushroom increased, the proportion of major naturally occurring *Z*-isomers of PTF and PT decreased. As for lycopene, the ratio of cis-lycopene increased with the addition of shii-take mushroom. The proportion of total cis-lycopene was increased to 40%, and 5-*Z*-lycopene accounted for 17.9% after heating with 50% shii-take mushroom. Similarly, the total content of PTF and PT hardly decreased, while lycopene decomposed as unstable cis-configuration formed.

Manufacturing process has an effect on the bioaccessibility of carotenoids [13]. Table 4 and Table 5 illustrate the bioaccessibility of PTF, PT, and lycopene in tomato shii-take mushroom mixtures after heating at 80 °C for 1 h. As shown in the tables, the bioaccessibility of PTF and PT (over 20%) was far more than that of lycopene (1.7%) without the addition of shii-take mushroom. This can be partially explained by the high micellization efficiency due to the natural existing cis-isomers of PTF and PT [9], while all-*E*-lycopene forms crystals in chromoplasts.

In the case of PTF, the bioaccessibility of all-*E*-PTF was 29.4%, slightly higher than that of *Z*1,2-PTF. Paula et al. [39] also found that the bioaccessibility of all-*E*-PTF and PT was slightly higher than that of their cis-isomers, which is illustrated by the molecule competition for incorporation into micelles resulted by the higher concentrations [28]. Moreover, there was no positive effect on the bioaccessibility of PTF and PT with the addition of shii-take mushroom after thermal treatment. The bioaccessibility of total PTF (as illustrated in Table 4) hardly changed, while it decreased significantly (*p* < 0.05) for PT (as illustrated in Table 5) as all-*E*-PT formed. As for lycopene, the bioaccessibility of lycopene isomers almost decreased in the order: 13-*Z* > 9-*Z* > 5-*Z* > All-*E*. *Z*-lycopene are more easily transferred into the mixed micelles than all-*E*-lycopene, which are more likely to aggregate and crystallize [18]. With the increasing addition of shii-take mushroom, the bioaccessibility of total lycopene increased significantly due to the formation of cis-lycopene. Even though there seems to be a negative effect on the bioaccessibility of PT and PTF with the addition of shii-take mushroom, their bioaccessibilities were still much higher than that of lycopene. Thus, it is necessary to monitor the isomers of colorless carotenoid and lycopene with food-containing natural catalysts to forecast their expected isomeric profiles in foods processing.

## 4. Conclusions

This study investigated the isomerization and degradation of lycopene, PTF, and PT in tomato pulp with sulfur-containing compounds. Sulfur-containing compounds had the effect on the isomerization of lycopene, PTF, and PT, and increasing the heating temperature, time, and concentration of DMTS and AITC could promote the isomerization reaction effectively. Meanwhile, the isomers of PTF and PT had good thermal stability. Moreover, the bioaccessibility of lycopene could increase significantly with the addition of shii-take mushroom containing sulfur compounds, although this had no positive effect on that of PTF and PT. However, the bioaccessibility of PTF and PT were still far higher than that of lycopene. According to the study, cooking or processing tomatoes with the foods containing sulfur-containing compounds could improve the absorbency of total carotenoids. These findings can provide a reference to forecast their expected isomeric profiles in tomato and other carotenoid-containing manufacturing. 

## Figures and Tables

**Table 1:** Identification of isomers of phytofluene, phytoene, and lycopene.

Carotenoids	Retion Time (min) Observed	λmax (nm)	%III/II	%III/II	Q-Ratio	Q-Ratio
			Observed	Reported	Observed	Reported
*Z*1-PTF	9.63	332,347,364	70.51	52.2 ^a^, 68.8 ^b^	-	-
*Z*2-PTF	9.85	332,347,364	78.64	82.2 ^a^, 70.2 ^b^	-	-
*Z*3-PTF	10.44	332,347,364	80.65	89.3 ^a^, 100 ^b^	-	-
All-*E*-PTF	11.09	331,347,364	82.61	91.4 ^a^, 86.5 ^b^	-	-
*Z*4-PTF	11.59	331,347,364	78.91	90.3 ^a^, 77.5 ^b^	-	-
*Z*1-PT	8.33	286	-	-	-	-
15-*Z*-PT	8.60	286	9.09	-	-	-
All-*E*-PT	9.12	286	14.35	19.05 ^a^	-	-
13-*Z*-LYC	29.61	360,439,464,496	-	-	0.58	0.56 ^c^
9-*Z*-LYC	31.71	360,439,465,497	-	-	0.14	0.13 ^a^
All-*E*-LYC	34.65	361,446,472,503	-	-	0.06	0.06 ^d^
5-*Z*-LYC	35.07	360,446,472,503	-	-	0.06	0.08 ^a^

^a^ Meléndez, et al. [29], 2013; ^b^ Lu, et al. [30], 2018; ^c^ Murakami, et al. [31], 2018; ^d^ Schierle, et al. [15], 1997.

**Table 2:** Effects of different sulfur-containing compounds on PTF and PT.

Types	Remaining PTF (%)	Proportion of PTF Isomers (%)				Remaining PT (%)	Proportion of PT Isomers (%)		
		*Z*1,2-PTF	*Z*3-PTF	All-*E*-PTF	*Z*4-PTF		*Z*1-PT	15-*Z*-PT	All-*E*-PT
Control	104.5 ± 3.6 ^a^	76.5 ± 1.1 ^a^	—	23.5 ± 1.1 ^abc^	—	98.8 ± 4.2 ^a^	—	100 ^a^	—
MITC	98.3 ± 0.3 ^a^	28.0 ± 0.3 ^bc^	13.1 ± 1.6 ^b^	28.4 ± 4.6 ^a^	30.5 ± 3.2 ^c^	97.0 ± 3.4 ^a^	—	95.7 ± 2.1 ^a^	4.3 ± 2.1 ^b^
BITC	97.14 ± 2.5 ^a^	31.8 ± 1.1 ^b^	13.4 ± 0.5 ^b^	23.6 ± 0.1 ^abc^	31.2 ± 1.3 ^bc^	99.9 ± 2.1 ^a^	—	94.0 ± 1.3 ^a^	6.0 ± 1.3 ^b^
AITC	101.6 ± 0.8 ^a^	28.1 ± 0.2 ^bc^	14.3 ± 0.7 ^ab^	25.1 ± 0.1 ^ab^	32.5 ± 0.3 ^abc^	99.8 ± 0.4 ^a^	—	93.5 ± 0.1 ^a^	6.5 ± 0.1 ^b^
DADS	95.7 ± 0.1 ^a^	27.8 ± 1.1 ^bc^	18.2 ± 0.1 ^a^	17.2 ± 0.5 ^bc^	36.8 ± 0.5 ^abc^	103.1 ± 0.3 ^a^	6.7 ± 0.0 ^b^	71.6 ± 0.1 ^b^	21.7 ± 0.2 ^a^
DATS	98.4 ± 3.5 ^a^	27.9 ± 1.2 ^bc^	17.1 ± 0.3 ^ab^	15.2 ± 0.1 ^bc^	39.8 ± 1.6 ^ab^	106.5 ± 2.1 ^a^	8.1 ± 1.2 ^ab^	64.1 ± 4.2 ^bc^	27.8 ± 2.9 ^a^
DMTS	94.5 ± 0.2 ^a^	26.9 ± 0.1 ^c^	17.3 ± 0.3 ^ab^	14.8 ± 0.3 ^c^	40.9 ± 0.2 ^a^	102.9 ± 0.8 ^a^	11.8 ± 0.1 ^a^	59.9 ± 0.6 ^c^	28.3 ± 0.5 ^a^

Data followed by different letters in same column are significantly different (*p* < 0.05). MITC: Methyl isothiocyanate; BITC: butyl isothiocyanate; AITC: allyl isothiocyanate; DADS: diallyl disulfide; DATS: diallyl trisulfide; DMTS: dimethyl trisulfide; Remaining PTF: PTF content in tomato pulp after thermal treatment compared to that of content in the raw tomato pulp; remaining PT: PT content in tomato pulp after thermal treatment compared to that of content in raw tomato pulp.

**Table 3:** Effects of different sulfur-containing compounds on lycopene.

Types	Remaining Lycopene (%)	Proportion of Lycopene Isomers (%)					
		All-*E*-LCY	Total-*cis*	5-*Z*	9-*Z*	13-*Z*	Others-*cis*
control	98.6 ± 1.3 ^a^	78.5 ± 0.6 ^a^	21.5 ± 0.6 ^d^	5.4 ± 0.8 ^d^	1.8 ± 0.1 ^e^	8.7 ± 0.1 ^d^	5.7 ± 0.0 ^b^
MITC	84.6 ± 0.4 ^bc^	52.9 ± 0.5 ^b^	47.1 ± 0.5 ^c^	22.0 ± 0.6 ^a^	6.4 ± 0.4 ^d^	9.7 ± 0.6 ^cd^	9.0 ± 0.1 ^ab^
BITC	86.3 ± 0.2 ^b^	55.3 ± 2.6 ^b^	44.7 ± 2.6 ^c^	14.3 ± 0.6 ^c^	7.8 ± 0.5 ^bcd^	9.7 ± 0.9 ^cd^	12.9 ± 3.5 ^ab^
AITC	81.5 ± 2.1 ^bc^	49.2 ± 0.4 ^bc^	50.8 ± 0.4 ^bc^	19.7 ± 0.2 ^ab^	8.7 ± 0.1 ^bc^	10.6 ± 0.1 ^cd^	11.8 ± 0.1 ^ab^
DADS	83.9 ± 0.9 ^bc^	57.1 ± 2.1 ^b^	42.9 ± 2.1 ^c^	15.3 ± 0.6 ^bc^	7.0 ± 0.6 ^cd^	11.3 ± 0.1 ^bc^	9.3 ± 0.5 ^ab^
DATS	84.5 ± 0.8 ^bc^	42.1 ± 1.4 ^cd^	57.9 ± 1.4 ^ab^	21.7 ± 0.5 ^a^	9.9 ± 0.3 ^b^	13.1 ± 0.4 ^ab^	13.2 ± 0.2 ^ab^
DMTS	84.1 ± 0.7 ^bc^	39.8 ± 1.3 ^d^	60.2 ± 1.3 ^a^	20.6 ± 1.9 ^a^	12.2 ± 0.5 ^a^	14.0 ± 0.1 ^a^	13.4 ± 0.4 ^a^

Data followed by different letters in same column are significantly different (*p* < 0.05). Remaining lycopene: the lycopene content in tomato pulp after thermal treatment compared to that of content in raw tomato pulp.

**Table 4:** Bioaccessibility (%) of phytofluene (PTF) and phytoene (PT) in tomato with different addition of shii-take mushroom.

Shii-Take Mushroom (%)	PTF (%)					PT (%)			
	Total-PTF	*Z*1,2-PTF	*Z*3-PTF	All-*E*-PTF	*Z*4-PTF	Total-PT	*Z*1-PT	15-*Z*-PT	All-*E*-PT
0	23.25 ± 0.5 ^ab^	20.36 ± 0.8 ^ab^	—	29.41 ± 0.3 ^a^	—	25.41 ± 2.1 ^a^	—	25.41 ± 2.1 ^a^	—
10%	21.61 ± 0.4 ^b^	19.53 ± 0.3 ^b^	—	25.98 ± 1.2 ^a^	17.03 ± 1.3 ^a^	25.37 ± 0.9 ^a^	—	25.45 ± 0.9 ^a^	23.32 ± 1.9 ^a^
20%	24.39 ± 0.7 ^a^	22.67 ± 1.1 ^ab^	17.07 ± 1.2 ^a^	28.03 ± 0.7 ^a^	19.90 ± 1.5 ^a^	24.82 ± 0.5 ^a^	12.47 ± 1.7 ^ab^	25.27 ± 0.6 ^a^	24.15 ± 0.8 ^a^
30%	23.58 ± 1.4 ^ab^	21.80 ± 2.1 ^ab^	17.57 ± 1.4 ^a^	31.83 ± 3.1 ^a^	18.48 ± 0.5 ^a^	23.96 ± 0.1 ^ab^	7.93 ± 0.1 ^b^	25.24 ± 0.1 ^a^	22.46 ± 1.1 ^a^
40%	24.96 ± 0.6 ^a^	24.06 ± 0.1 ^a^	17.91 ± 0.1 ^a^	29.09 ± 0.8 ^a^	23.13 ± 0.9 ^a^	22.04 ± 0.5 ^b^	6.97 ± 0.2 ^b^	24.30 ± 0.4 ^a^	16.75 ± 0.1 ^b^
50%	23.96 ± 0.2 ^ab^	23.34 ± 0.9 ^ab^	21.45 ± 2.6 ^a^	27.28 ± 0.9 ^a^	21.41 ± 0.3 ^a^	19.18 ± 0.8 ^c^	17.87 ± 0.3 ^a^	21.75 ± 1.2 ^a^	17.36 ± 0.1 ^b^

Data followed by different letters in same column are significantly different (*p* < 0.05).

**Table 5:** Bioaccessibility (%) of lycopene in tomato with different addition of shii-take mushroom.

Shii-Take Mushroom (%)	Lycopene (%)				
	Total Lycopene	All-*E*	5-*Z*	9-*Z*	13-*Z*
0%	1.71 ± 0.2 ^e^	0.74 ± 0.1 ^e^	4.83 ± 0.4 ^c^	7.48 ± 1.2 ^b^	8.77 ± 1.1 ^b^
10%	4.00 ± 0.1 ^d^	2.78 ± 0.2 ^d^	8.12 ± 0.7 ^bc^	7.81 ± 0.2 ^b^	9.04 ± 1.2 ^b^
20%	4.42 ± 0.2 ^cd^	3.22 ± 0.1 ^cd^	7.81 ± 0.8 ^bc^	9.11 ± 1.6 ^b^	7.88 ± 0.2 ^b^
30%	5.42 ± 0.4 ^c^	3.69 ± 0.2 ^c^	9.06 ± 0.8 ^bc^	9.98 ± 0.8 ^ab^	9.24 ± 0.1 ^ab^
40%	6.67 ± 0.4 ^b^	4.43 ± 0.3 ^b^	9.49 ± 0.5 ^ab^	11.58 ± 1.8 ^ab^	9.37 ± 1.1 ^ab^
50%	8.03 ± 0.5 ^a^	6.30 ± 0.2 ^a^	11.86 ± 1.4 ^a^	13.81 ± 1.2 ^a^	12.89 ± 1.4 ^a^

Data followed by different letters in same column are significantly different (*p* < 0.05).
